# 

**Published:** 1849-12

**Authors:** 


### CONTENTS

### OF NO. VI

### ORIGINAL COMMUNICATIONS.

### ESSAYS AND CASES.

ART. I.—On the Use of the Trephine in Injuries of the Head. By Dr. THOMAS LIPSCOMB, of Shelbyville, Tenn. - 461

ART. II —A Case of Facial Palsy consequent upon Otitis. By W. H. COBB, M. D., of the Louisville Marine Hospital. 469

ART. III.—Natural History of Cinchona, (Peruvian Bark.) By Dr. WEDDELL. Translated from the Journal de Pharmacie et de Chemie, for Sept. 1849, by BENJAMIN SILLIMAN, Jr., M. D., Prof. of Medical Chemistry and Toxicology in the Medical Department of the University of Louisville. - - - - - - 472

ART. IV.—Practical Medicine and Surgery. By T. S. BELL, M. D., of Louisville, Kentucky, - - - 484

### REVIEWS.

ART. V—.A Treatise on the Diseases of the Bones. By EDWARD STANLEY, F. R. S., President of the Royal College of Surgeons of England, and Surgeon to St. Bartholomew's Hospital, - - - - 493

ART. VI.—On the Pathology and Treatment of Epidemic Cholera. By SAMUEL ANNAN, M. D., Professor of the Theory and Practice of Medicine, in Transylvania University. - - - - - - - 506

ART. VII.—The Practice of Surgery: embracing Minor Surgery, and the application of Dressings, etc., etc.. By JOHN HASTINGS, M. D., U. S. N., Fellow of the College of Physicians of Philadelphia, Lecturer on Surgical Anatomy and Operative Surgery, etc., etc. - - 519

SELECTIONS FROM AMERICAN AND FOREIGN JOURNALS.

On Functional Disease of the Liver, associated with Uterine Derangement. - - - - - 521
On the Treatment of Obstinate Dyspeptic Symptoms. - 523
On a Solution of Iodine in Oil. - - - - 524
Use of Sulphate of Iron in Chancre and Gonorrhea. - 525
On the Use of Coffee in Infantile Cholera. - - - 526
On the Use of Iodide of Potassium in Chronic Lead Poisoning. 527
Cases of Poisoning by Chloride of Zinc. - - - 529
On the Presence of Uric Acid in the Kidneys, as a Sign of a Child having been born alive. - - - 530
Treatment of Rigidity of the Os Uteri. - - - 531
On the Signs of Diseased Heart afforded to the Hand laid over the Precordia. - - - - - - 532
Treatment of Dropsy after Scarlet Fever. - - - 534
On the Employment of Cupping-Glasses to the Spine, in Intermittent Fever. - - - - - - 534
Table of the Pulses in Diseases of the Heart. - - 535
Fracture of the Neck of the Femur within the Capsule—Bony Union. - - - - - - 537
Microscopic Examination of the Contents of the Hepatic Ducts, with Conclusions founded thereon. - - 536
Compound Fractures, Amputations, &c. - - - 538

ORIGINAL INTELLIGENCE.

Medical Department of Louisville University, - - 543
Books Received, - - - - - - - 544
Errata. - - - - - - - - 545

